# ICPC-2-defined pattern of illnesses in a practice-based research network in an urban region in West Africa

**DOI:** 10.4102/phcfm.v1i1.3

**Published:** 2009-08-25

**Authors:** Olayinka O. Ayankogbe, Muriel A. Oyediran, David A. Oke, Surajudeen O. Arigbabu, Akin A. Osibogun

**Affiliations:** 1 Department of Community Health & Primary Care, University of Lagos, Nigeria; 2 Department of Medicine, University of Lagos, Nigeria; 3 Department of Surgery, University of Lagos, Nigeria

**Keywords:** practice-based, ICPC-2, illnesses, urban, West Africa

## Abstract

**Background:**

In optimising the health of individuals, families and communities, attention should be focused on the clinical processes at medical facilities based in the community. Networks of general and family practices offer this unique opportunity. In establishing the burden of diseases in a community, the traditional classification used is the International Classification of Diseases. This study uses the International Classification of Primary Care. The aim of the study was to document the pattern of illnesses presenting in general/family physician practices in a city in Nigeria.

**Method:**

A nine-item interviewer-administered questionnaire containing closed-ended questions was administered to 881 patients presenting at 67 private general/family practice clinics/hospitals in 15 local government areas of urban Lagos by trained general practitioners, using the ICPC-2 pager, which asks for socio-demographic information, reasons for the presentation, and the provisional diagnosis within a 24-hour period.

**Results:**

Children younger than five years accounted for 20.4% of those presenting, while 25- to 39-year-olds accounted for 44.4%. Geriatric patients (60 years and older) comprised 3.0%. Social classes 1 to 4 accounted for 36.8% of the patients, while social classes 5 to 8 accounted for 43.2%. Of all the patients, 18.7% earned less than 1 US$/day. The seven topmost reasons for visiting the medical practice/clinic/hospital were: General and unspecified 23.1%; pregnancy, child bearing and family planning 13.9%; respiratory problems 10.9%; problems related to the digestive system 9.6%; musculoskeletal 5.6%; Skin 4.4%; and neurological problems 4.2%.

**Conclusion:**

The skills of general/family practitioners in West Africa and on the rest of the continent should concentrate on general and unspecified illnesses, routine and emergency maternal and child care, and problems related to the respiratory, digestive, musculoskeletal, skin and neurological systems.

## INTRODUCTION

In optimising the health of individuals, families and communities, attention needs to be focused on clinical processes at health and medical facilities based in the community.^1, 2, 3^ Networks of general and family practices are part of the structures that offer a unique opportunity to do this.^3, 4^ This is because of their widespread distribution in the communities they serve, making them much more easily accessible than other secondary and tertiary centres like general and teaching hospitals, especially in the West African setting. In establishing the burden of disease in a community, the traditional classification used is the International Classification of Diseases (ICD). Until the mid-1970s, most morbidity data collected in primary care settings for statistics and research was classified using the ICD.^4^ This had the important advantage of gaining international recognition, thus aiding the comparability of data from different countries. However, the disadvantage was that the many symptoms and non-disease conditions that present in primary care were difficult to code using this classification, which was originally designed for application to mortality statistics, and with a disease-based structure.^4^ Quite a number of these disease conditions do not progress to full-blown ICD-compliant illnesses.

The Classification Committee of the World Organisation of National Colleges, Academies and Academic Associations of General Practitioners/Family Doctors (WONCA) (now the World Organization of Family Doctors (Wonca)) first met in 1972 in Melbourne at the time of its inauguration. Many of its members had already been corresponding for some years about morbidity classifications for general practice. The Committee agreed that it was time to design a classification specifically for primary care.^4^ Recognising the problems associated with the ICD and the need for an internationally recognised classification for general practice, the WONCA Classification Committee (now the Wonca International Classification Committee (WICC)) designed the International Classification of Primary Care (ICPC).

The ICPC, which was developed by the ICPC Working Party, broke new ground in the world of classification when it was published by WONCA in 1987.^3^ It enabled health care providers to classify three important elements of the health care encounter using a single classification: the reasons for encounter, diagnoses or problems, and the process of care. The problem orientation of the medical record and a linkage of encounters over time permit the classification of the episode from the beginning, starting with the RFE, to its conclusion with a more defined problem, diagnosis or disease.^3^

The new classification departed from the traditional chapter format of the International Classification of Disease (ICD), in which the axes of the chapters vary from body systems to aetiology^3^ and others. This mixture of axes creates confusion, since diagnostic entities can be classified into more than one chapter with equal logic, for example influenza in either the infections chapter or in the respiratory chapter, or in both. Instead of conforming to this format, the ICPC chapters are all based on body systems,^3^ following the principle that localisation has precedence over aetiology. The components that are part of each chapter permit considerable specificity for all three elements of the encounter, yet their symmetrical structure and frequently uniform numbering across all chapters facilitate usage even in manual recording systems. The rational and comprehensive structure of the ICPC is a compelling reason to consider the classification as a model for future international classifications.^3^

Since its publication, the ICPC has gradually received increasing international recognition as an appropriate classification for general/family practice and primary care, and has been used extensively in some parts of the world, notably in Europe^3, 5, 7^ and Australia.^3, 6^ The first version of the ICPC was published in 1987 and is referred to as ICPC-1. The 1998 version is referred to as ICPC-2. ICPC-2-E refers to a revised electronic version released in 2000. Subsequent revisions of ICPC-2 are also labelled with a release date. The ICPC is used when referring to the generic classification. Therefore, for a more accurate and relevant appreciation of the distribution and nature of diseases coming straight from the communities and presenting in these primary medical and health facilities, the International Classification of Primary Care has been developed.^7^

The International Classification of Primary Care (ICPC) has now been available to the family medicine community for two decades as the main ordering principle of its domain.^8^ The burden of disease in primary care settings in Europe^3, 7^ and Australia^3, 9^ has been ascertained using this unique classification.

However, little published work is available on the situation in sub-Saharan Africa.

This study therefore, as a baseline study, seeks to shed light on the burden and pattern of illnesses presenting in family physician primary care practices in Lagos, Nigeria using the ICPC-2 classification.

## METHOD

All patients presenting on a chosen day (24 hours) in 67 consenting clinics/hospitals out of 375 randomly selected clinics/hospitals (total 3 500) in the University of Lagos Teaching Hospital-linked Family Medicine Practice-based Research Network participated in the study. These practices were located in 15 local government areas that make up the urban Lagos State. A nine-item interviewer-administered questionnaire containing closed-ended questions asking about socio-demographic information, reasons for encounter, local diagnosis and reasons for encounter coding were administered on all 881 patients who attended the 67 clinics/hospitals on the chosen day. Clinics were outpatient clinics consisting of three to four rooms, while the hospitals were clinics with additional space of between five and 25 beds. Both the clinics and the hospitals run 24-hour services. All the practices run comprehensive general/family practice services.

The interviewers were qualified doctors (mostly general practitioners) recruited from each of the 67 private hospitals and subjected to a one-day training workshop to familiarise them with data-gathering methodologies using the ICPC-2 pager. They later trained the other doctors in their practices. The completed questionnaires were retrieved from the practices by the field workers at the end of the 24-hour data-gathering period. The ICPC-2 coding by the doctors was cross-checked by the principal investigator for validity and the correction of any errors. The questionnaires were analysed using the computer program EPIINFO, version 6.04b. The ICPC-2 categories were analysed along the lines of components and chapters using the ICPC-2 pager to identify rubrics.

## RESULTS

All 881 patients presenting within the 24-hour period of the study were interviewed. The respondents were distributed throughout all the local government areas that make up urban Lagos (29.6%, 6.1%, 16.4%, 17.5%, 3.9%, 4.0%, 2.7%, 1.9%, 4.1%, 7.1%, 3.3%, 2.8% and 0.6% in each of the local government areas respectively).

Table 1 shows the distribution according to age groups that patronise these practices. Most were under-fives (20.4%) and 25- to 39-year-olds (44.4%). Only 14.4% of the patients were young people (10 to 24 years), and only a further 3% were adolescents (15 to 19 years). The percentage of geriatric patients (60 years and above) was conspicuously low at 3.0%.

**TABLE 1:** Socio-demographic data of respondents presenting at the 67 clinics

	FREQUENCY	%
**AGE**		
< 5 years	180	20.4%
5–9 years	40	4.50%
10–14 years	20	2.3%
15–19 years	26	3.0%
20–24 years	80	9.1%
25–29 years	157	17.8%
30–34 years	147	16.7%
35–39 years	87	9.9%
40–44 years	43	4.9%
45–49 years	29	3.3%
50–54 years	25	2.8%
55-59 years	20	2.3%
60–64 years	14	1.6%
65–69 years	10	1.1%
70 and >	3	0.3%
**SEX**		
Male	389	44.1%
Female	492	55.1%
**MARITAL STATUS**		
Single	397	45.1%
Married	451	51.2%
Divorced/Separated	13	1.5%
Widow/Widower	17	2.1%
Live-in lover	1	0.1%
**CLASS, OCCUPATIONAL/PROFESSIONAL CATEGORIES** (after the United Kingdom 2001 socio-economic classifications)		
1. Higher managerial and professional	62	7
2. Lower managerial and professional	101	11.5
3. Intermediate	71	8.1
4. Small employers and own account workers	90	10.2
5. Lower supervisory and technical	85	9.7
6. Semi-routine	88	10
7. Routine	131	14.9
8. Never worked and long-time unemployed	76	8.6
9. Full time students and inadequately described occupations	177	20

**TOTAL**	**881**	**100**

### Sex distribution of respondents

Of the total number of patients, 44.1% were males and 55.9% were females, giving a male/female ratio of 1/1.2 (see Table 1). Nearly half (45.1%) were not married, while 51.2% were married and 2.1% were widowed (see Table 1). Table 1 also shows that there was an even distribution of patients along the spectrum of social classes and occupations. Social classes 1 to 4 accounted for 36.8% of the patients, while social class 5 to 8 accounted for 43.2%. Students and inadequately described occupations accounted for 19.7%.

Table 2 shows the monthly income of the respondents, and it was found that 18.7% of patients patronising urban private practices in Lagos were poor (earning less than 1 US$/day).

**TABLE 2:** Monthly income of respondents (in US$) (1 US$ = N130)

MONTHLY INCOME (in N)	(in US$)	FREQUENCY	%
<2000	< 15	69	10.2
2,001–3,999	16–30	55	8.5
4,000–5,999	31–4	8	12.5
6,000–7,999	47–61	83	12.2
8,000 and above	> 61	387	57

**TOTAL**		**679**	**100**

Table 3 shows the stated reasons why these patients visited the clinics and it was found that many (23.1%) visited because of general and unspecific illnesses.

**TABLE 3:** Reasons for encounter among 881 respondents

RFE	FREQUENCY	%
General and unspecified	204	23.1%
Blood, blood forming organs and immune mechanisms	2	0.2%
Digestive	85	9.6%
Eye	14	1.6%
Ear	14	1.6%
Cardiovascular	15	1.7%
Musculoskeletal	49	5.6%
Neurological	37	4.2%
Psychological	12	1.4%
Respiratory	96	10.9%
Skin	39	4.4%
Endocrine/metabolic and nutrition	6	0.7%
Urological	9	1.0%
Pregnancy, childbearing and family planning	123	13.9%
Female genital	34	3.9%
Male genital	8	0.9%
Social problems	7	0.8%
Process codes	127	14.5%

**TOTAL**	**881**	**100.0%**

The seven topmost reasons why these patients visit these private clinics/hospitals were the following, in descending order of frequency: General and unspecified 23.1%; pregnancy, child bearing and family planning 13.9%; respiratory problems 10.9%; problems related to the digestive system 9.6%; musculoskeletal 5.6%; skin 4.4%; and neurological problems 4.2%.

Other, less prominent reasons for encounter include female genital problems (3.9%). while the least of the presenting problems concern cardiovascular disease (1.7%) and psychological disease (1.4%).

## DISCUSSION

We present the first published findings of the distribution of illnesses and diseases presenting in private general practices in Lagos, Nigeria, using the ICPC-2, an essential tool in the practice records of general practitioners.^9^

The use of the ICPC-2 for the classification of diseases presenting in general/family practices has been on the increase in different parts of the world.^3, 5, 7^ This study confirms that it can also be used in Africa to map out the majority of illnesses presenting at general/family practices. The ICPC-2 is also relevant for practicing physicians, as it helps to document the trend and burden of illnesses for planning purposes, not only in the physician's practice, but also in the state-wide network and in the country of practice as a whole. This can be used as a powerful advocacy tool in relation to policy makers, both public and private. For example, the study confirms the low patronage of these practices by adolescents and geriatric patients. This calls for research on the reasons why this is so and the suggestion of workable interventions. The surveillance of epidemic diseases as they present to these practices can also be carried out using this simple classification. Continuous analysis of the ICPC-2 data generated by these practices will aid in the very early detection of dangerous epidemics and disease trends, which will lead to early intervention and control.

There is very little published data on the continent with which to compare these findings. This work might serve as a baseline for further studies in Africa.

There is low patronage by adolescents and geriatrics, and high patronage by children under five, as can be expected in developing Africa. It is pertinent to note that the social classes of the presenting patients cut across all social strata. This is a significant finding, as private general practices in Nigeria and in Africa are usually expected to be patronised only by the upper social classes. To buttress this fact, it was also significant to note that almost 20% of the respondents patronising these private general practices fell below the poverty line.

A large percentage of the illnesses are general and unspecified. There is high patronage for pregnancy, child-bearing and family planning, as expected in a developing country. There is an unexpected distribution of illnesses and diseases when compared to practices in Europe, America and Australasia, with respiratory, digestive, skin and musculoskeletal problems taking centre stage. Almost the same distribution was found as in general practices in other developing countries, such as Pakistan^10^ and Sri Lanka.^11^ In a university primary care clinic in Saudi Arabia, the morbidity trends varied little.^12^ In the Seychelles, which belongs to the African continent, the commonest illness presenting was hypertension.^13^ This is a departure from what is usually expected. The health system and health care delivery in Seychelles are far advanced compared to other African health service delivery.^13^ The Seychelles health system is patterned strictly according to the British system.^13^ Therefore, one might expect this trend in other developing countries and in Africa in the future, as the general socioeconomic circumstances improve.

### Ethical considerations

Ethics approval for the study was obtained from the Central Research and Ethics Committee of the College of Medicine, University of Lagos. Verbal and written consent was obtained from each of the patients interviewed by the doctors collecting the data at the clinics/hospitals.

### Limitations of the study

Only 67 (17.8%) of the 375 targeted, randomly selected practices took part in the study. This is 67/3 500 (0.01%) of the total estimated number of 3 500 urban practices in the state. It is possible that this low number introduced bias because of the fact that it does not adequately represent the total population of practices. However, there is an approximately even spread of practices in the local government areas (see results).

### Conclusion

The fact that many common illnesses, chronic diseases and preventive treatments are dealt with in general practice also shows the necessity to include training in family medicine in the undergraduate curriculum of all medical schools in West Africa and in the rest of the continent. Undergraduate and postgraduate training in family medicine in West Africa should concentrate more on the identification, diagnosis and effective treatment of general and unspecified illnesses, routine and emergency maternal and child care, respiratory problems, problems related to the digestive system, musculoskeletal problems, skin problems and neurological problems, as these are the most common problems identified by this study.

These results obviously also have enormous implications for health policy makers, the National Health Insurance Scheme, health maintenance organisations, postgraduate colleges in family medicine, private medical practitioners and for the family physicians themselves.
